# Prevalence of Self-reported Cognitive Impairment Among Arab American Immigrants in the United States

**DOI:** 10.1093/geroni/igaa058

**Published:** 2020-11-24

**Authors:** Florence J Dallo, Tiffany B Kindratt, Laura Zahodne

**Affiliations:** 1 Department of Public & Environmental Wellness, Oakland University, Rochester, Michigan, US; 2 Public Health Program, Department of Kinesiology, College of Nursing and Health Innovation, University of Texas at Arlington, US; 3 Department of Psychology, University of Michigan, Ann Arbor, US

**Keywords:** Disability, Immigrant, Middle Eastern and North African, Nativity status

## Abstract

**Background and Objectives:**

Studies evaluating self-reported cognitive impairment among Arab American immigrants have not been conducted. Our objective was 2-fold: (a) to estimate and compare the age- and sex-adjusted prevalence of self-reported cognitive impairment between Arab American immigrants and U.S.- and immigrant non-Hispanic Whites, non-Hispanic Blacks, Hispanics and non-Hispanic Asians and (b) to examine associations between race, ethnicity, nativity status, and cognitive impairment among Arab American immigrants and non-Hispanic Whites (U.S.- and foreign-born) after controlling for explanatory factors.

**Research Design and Methods:**

We used 18 years (2000–2017) of National Health Interview Survey data (*n* = 228 985; ages ≥ 45 years). Weighted percentages, prevalence estimates, and multivariable logistic regression models were calculated.

**Results:**

The age- and sex-adjusted prevalence of self-reported cognitive impairment was significantly higher among Arab American immigrants (9.7%) compared to U.S.-born and non-Hispanic White immigrants (~7.4%).

**Discussion and Implications:**

This is the first study to indicate that ethnic disparities in self-reported cognitive impairment may extend to Arab American immigrants. Additional studies need to be conducted to better understand the prevalence of cognitive impairment.

**Translational Significance: ** Arab American immigrants may carry a higher burden of cognitive impairment compared to U.S.-born and White immigrants.

In the United States, the overall prevalence of self-reported cognitive impairment (hereafter referred to as cognitive impairment) was 6.7% in 2015 among individuals ages 60 or older (1). This varied by race, ethnicity, and nativity status. Non-Hispanic Blacks had higher estimates (9.2%) compared to Hispanics (8.7%), Asians (5.3%), and non-Hispanic Whites (6.%). With regard to nativity status, U.S.-born non-Hispanic Blacks had a higher prevalence of dementia compared with non-Hispanic Black immigrants (2). However, Hispanic and non-Hispanic White immigrants had a higher prevalence of dementia compared with their U.S.-born counterparts (2). Whites are a heterogeneous group, defined by the federal government as individuals from Europe, the Middle East and North Africa (hereafter, individuals from the Middle East and North Africa are referred to as Arab American) (3). Given that Arab Americans do not have a separate ethnic identifier in the Census or health surveys (either at the national or state level), their profile is masked under the White category; therefore, any variations in morbidity and mortality due to cognitive impairment are not distinguishable.

While research is available to demonstrate that Arab Americans, especially immigrants, report higher levels of physical and self-care disabilities compared to other groups (4,5), studies focused on cognitive impairment among Arab Americans have not been conducted. Among these and similar studies, there are 2 critical points to note. First, the emerging health pattern when comparing U.S.- to Arab American immigrants (6) is not consistent with the “immigrant health paradox” (7). That is, Arab American immigrants demonstrate a higher burden of disease than the U.S.-born, which is generally not the case for other racial and ethnic groups. Second, the 3 studies on disability used data from the American Community Survey. The current study analyzes data from the National Health Interview Survey (NHIS), the only national health study where Arab Americans can be disaggregated from the non-Hispanic White population.

The Arab American population in the United States is increasing. According to the U.S. Census, it has increased from 1.2 million in 2000 (8) to 1.7 million in 2010 (9). According to Zogby, the more accurate number is 3 times that reported by the U.S. Census (10)—approximately 3.7 million Arab Americans (11). Of these, roughly 55% (2 035 000) are immigrants (12). Elucidating the health of immigrants will help deploy appropriate resources to prevent or delay the onset of disease. To fulfill this goal, it is important to disaggregate Arab Americans from the White category, so that resources, prevention efforts, and policy decisions are tailored to this minority, immigrant, underserved, and often invisible population.

Estimating the prevalence of cognitive impairment is important for the reasons described above and for several other reasons. First, the current study also is a response to a recent paper that makes a strong case that better understanding the burden of cognitive impairments among Arab Americans will add to the discourse on health disparities (13). The authors state, “studying cognitive health issues among older Arab Americans provides an innovative opportunity to advance knowledge about causes and consequences of Alzheimer’s disease disparities and refine understanding of factors linked to immigrant health in the United States.” Second, the current study will provide national baseline estimates of cognitive impairment so that other research, interventions, and resources can be allocated to the populations that need it most. Lastly, a majority of health research focuses on adolescents and adults (14,15). There is a dearth of aging research on Arab Americans (13).

This study has 2 objectives: (a) to estimate and compare the age- and sex-adjusted prevalence of cognitive impairment among Arab Americans aged 45 or older compared to the majority population (U.S.-born non-Hispanic Whites), as well as non-Hispanic Black, Hispanic, and non-Hispanic Asian immigrants; and (b) to examine the associations between race, ethnicity, nativity status, and cognitive impairment when controlling for potential social, economic, and health factors.

## Research Design and Methods

### Data Source

The study sample comprised 18 years (2000–2017) of cross-sectional data from the NHIS. The NHIS is a nationally representative survey that utilizes a multistage probability-based sampling design to gather various characteristics of U.S. adults and children. African American, Hispanic, and Asian populations are oversampled (16). Face-to-face interviews (English and Spanish languages) occur in participants’ homes using a computer-assisted personal interviewing system. Weights are used to determine nationally representative estimates. In this study, the annual sampling weights were compiled and divided by 18 to reflect the total number of annual surveys compiled (16). More details on the NHIS design and analytical procedures are reported on their website (16).

### Participants

The total unweighted sample included 1 689 829 persons from 676 511 families and 662 272 households. Of the 1 689 829 persons interviewed, 546 922 individuals completed the sample adult questionnaire. All questions asked at the family level are linked with individual participants who completed the sample adult questionnaire. The sample for this study was limited to adults aged 45 and older who answered questions about nativity status (U.S.-born or foreign-born), race (White, Black, Asian), and ethnicity (Hispanic or non-Hispanic). The inclusion criterion was age 45 or older, because signs of cognitive impairment have been shown among individuals in their late 40s and early 50s (17), and to parallel one study that included an age cutoff of 45 years or older (18). We used this cutoff so that our findings would not be limited to comparisons of studies that included only those 65 years of age or older. The final sample size included 228 985 adults (U.S.-born = 189 129 and foreign-born = 39 856).

### Measures

#### Independent variables

The independent variable is a combined measure of ethnicity, race, and nativity status. Participants were asked whether they were Hispanic or Latino/a and provided flashcards to select their race (White, Black, American Indian/Alaskan Native, Asian, other) (16). Participants were asked whether they were born in one of the 50 states, Washington DC, on a military base overseas, or on a U.S. territory. Foreign-born persons were asked about their citizenship. All participants who were not born in the United States or in a U.S. territory were asked “in what country were you born,” and responses for each individual country were grouped into 10 world regions within the publicly available data files (Asia, Central America & Caribbean Islands, Europe, India subcontinent, Mexico, Middle East, Russia, South America, Southeast Asia, and the United States) (16). Responses to race, ethnicity, and nativity status questions were combined to compare U.S.-born non-Hispanic Whites to foreign-born non-Hispanic Blacks, Hispanics, non-Hispanic Asians, and Arab Americans. Because the NHIS collects race and ethnicity data based on the 1997 Office of Management and Budget Classification (3), there is no classification for Arab Americans. Respondents who indicated they were born in the “Middle East” region were categorized as Arab American immigrants (19). In other words, our independent variable is a combined measure of race, ethnicity, and nativity status to make comparisons between Arab American immigrants and (a) U.S-.born non-Hispanic Whites and (b) immigrant non-Hispanic Whites, non-Hispanic Blacks, Hispanics, and non-Hispanic Asians.

#### Dependent variable

The dependent variable in this study was self-reported cognitive impairment, which was asked at the family level and linked to individual responses for each sample adult. Each participant (or a reference person who served as proxy) was asked whether they were limited by difficulty remembering or because of periods of confusion (yes, no).

#### Covariates

Covariates were selected based on identified risk factors for cognitive impairment and previous studies (17,18). Demographic variables examined were age (mean; 45–54, 55–64, 65–74, 75+ years), sex (male, female), marital status (never married, married/live with partner, divorced/widowed/separated), and living situation (living alone, living with others). Socioeconomic variables examined were education (less than high school [HS], HS or GED, some college or associate degree, and Bachelor’s degree or higher), income based on the U.S. poverty thresholds (<200% and ≥200%), and current employment (employed or unemployed). Comorbidities examined were diabetes (yes or no), cardiovascular disease (yes or no), obesity (normal weight, overweight, obese), and serious psychological distress (yes or no). Behavioral risk factors examined were alcohol drinking status (current drinker, nondrinker), smoking status (current smoker, nonsmoker), and level of physical activity (<5 times per week, ≥5 times per week). Among immigrant adults, length of time living in the United States (<15 years, ≥15 years) and citizenship (yes, no) were examined as acculturation proxies based on previous studies (19–23).

### Analysis

Weighted column percentages and standard errors were obtained to report demographic characteristics, socioeconomic status, comorbidities, and behavioral risk factors among U.S.-born and immigrant adults. Acculturation proxies were reported for immigrant adults only. The age- and sex-adjusted prevalence of cognitive impairment indicators was obtained for U.S.-born non-Hispanic Whites and immigrant non-Hispanic Whites, non-Hispanic Blacks, Hispanics, non-Hispanic Asians, and Arab Americans. Multivariable logistic regression was used to determine the association between the combined measure of race, ethnicity, and nativity (independent variable) and cognitive impairment indicators (dependent variable) while controlling for potential confounding and mediating variables. Multilevel adjustment of covariates was conducted to adjust for the following: sex and age (Model 2); Model 2 plus marital status and living situation (Model 3); Model 3 plus education, income, and employment (Model 4); Model 4 plus comorbidities (Model 5), Model 5 plus behavioral risk factors (Model 6), and acculturation proxies among immigrant adults (Model 7). Immigrant Arab Americans were compared to both U.S.-born and immigrant non-Hispanic Whites. We conducted 2 sensitivity analyses (see Supplementary Tables 1 and 2) using our crude and multivariable logistic regression models to examine estimates among different age groups. We limited the sample to adults (a) aged 55 and older and (b) aged 65 and older.

Statistical analysis was completed using SAS 9.4. SAS survey procedures were used to account for the complex sample design. Adjustments were made to stratum and primary sampling unit variables used for variance estimation due to different sample designs (2000–2005, 2006–2014, and 2015–2017). More information on the changes in sample designs and recommended procedures for combining different designs are reported elsewhere (16).

This study was approved as exempt under criteria 45 CFR 46.104(d) by the University of Texas Southwestern Medical Center Human Research Protection Program/Institutional Review Board.

## Results

### Descriptive Results

Selected characteristics of the sample are reported in Table 1. The mean age of Arab American immigrants was older (59.1 years) than non-Hispanic Black (56.9 years), Hispanic (57.9 years), and non-Hispanic Asian (58.6 years) immigrants but younger than U.S.-born and non-Hispanic White immigrants (61.3 years and 63.2 years, respectively) (*p* < .0001). Arab American immigrants were more likely to be male (53.6%) and have a bachelor’s degree or higher (47.5%) compared to all other immigrant adults and U.S.-born non-Hispanic Whites (*p* < .0001). While the prevalence of diabetes and obesity was highest among Hispanic immigrants (18.4% and 33.8%, respectively) and the prevalence of cardiovascular disease was highest among U.S.-born non-Hispanic Whites (51.2%) compared to other groups, the prevalence of serious psychological distress was markedly higher among Arab American immigrants (8.2%) compared to non-Hispanic Asian (2.0%), Hispanic (4.8%), non-Hispanic Black (1.8%), non-Hispanic White (3.3%) immigrants, and U.S.-born non-Hispanic Whites (3.3%). Arab American immigrants were more likely to be current smokers (14.7%) than their immigrant counterparts (non-Hispanic Whites = 13.3%; Hispanics = 10.9%; non-Hispanic Asians = 8.6%; and non-Hispanic Blacks = 6.4%). Arab American immigrants were less likely to be citizens compared to all other immigrant adults (*p* < .0001).

**Table 1.** Selected Characteristics of Sample for U.S.-Born and Immigrant Adults, NHIS 2000–2017, *N* = 228 985

	U.S.-Born	Immigrants					
	NH Whites, *n* = 189 129	NH Whites, *n* = 6048	NH Blacks, *n* = 3187	Hispanics, *n* = 21 367	NH Asians, *n* = 8467	Arab Americans, *n* = 787	
Variable	Weighted % (*SE*)	Weighted % (*SE*)	Weighted % (*SE*)	Weighted % (*SE*)	Weighted % (*SE*)	Weighted % (*SE*)	*p*-Value
Age							
Mean age (*SD*)	61.3 (0.05)	63.2 (0.19)	56.9 (0.23)	57.9 (0.11)	58.6 (0.17)	59.1 (0.49)	<.0001
45–54 y	35.6 (0.18)	30.8 (0.79)	53.5 (1.25)	48.6 (0.56)	44.8 (0.70)	44.1 (2.65)	<.0001
55–64 y	27.1 (0.14)	24.8 (0.74)	24.2 (1.20)	25.6 (0.44)	26.4 (0.59)	26.4 (2.07)	
65–74 y	20.3 (0.13)	22.0 (0.62)	14.7 (0.81)	16.0 (0.32)	18.5 (0.50)	17.0 (1.54)	
75+ y	17.0 (0.15)	22.3 (0.66)	7.7 (0.57)	9.8 (0.26)	10.3 (0.49)	12.5 (1.22)	
Male sex	47.2 (0.15)	44.1 (0.74)	47.6 (1.16)	48.0 (0.47)	46.3 (0.65)	53.6 (2.17)	<.0001
Marital status							<.0001
Never married	5.8 (0.07)	4.3 (0.28)	10.5 (0.61)	7.1 (0.21)	3.8 (0.24)	3.6 (0.71)	<.0001
Married/live with partner	68.7 (0.18)	68.1 (0.69)	59.8 (1.06)	68.2 (0.46)	79.2 (0.56)	75.3 (1.60)	
Divorced/widowed/separated	25.9 (0.15)	27.6 (0.67)	29.7 (0.91)	24.6 (0.41)	17.0 (0.48)	21.2 (1.38)	
Live alone (% yes)	22.6 (0.16)	23.9 (0.60)	18.8 (0.73)	13.3 (0.30)	10.6 (0.41)	14.4 (1.09)	
Education							<.0001
Less than high school	11.7 (0.13)	15.4 (0.59)	17.6 (0.98)	52.4 (0.55)	16.1 (0.57)	18.0 (1.60)	
High school/GED	30.6 (0.18)	25.2 (0.70)	28.0 (1.07)	20.7 (0.38)	20.5 (0.61)	19.4 (1.79)	
Some college/associate’s	28.2 (0.15)	24.2 (0.63)	26.0 (1.06)	14.5 (0.32)	16.6 (0.50)	15.0 (1.56)	
Bachelor’s or higher	29.6 (0.24)	35.2 (0.77)	28.4 (1.10)	12.4 (0.35)	46.8 (0.78)	47.5 (2.83)	
Poverty level (200% or greater)	70.9 (0.20)	66.2 (0.95)	57.3 (1.53)	42.0 (0.53)	61.6 (0.74)	55.5 (2.34)	<.0001
Employed (% no)	49.9 (0.20)	54.0 (0.83)	33.4 (1.14)	46.5 (0.51)	42.0 (0.75)	47.4 (2.15)	<.0001
Diabetes (% yes)	12.2 (0.09)	10.5 (0.52)	17.6 (0.91)	18.4 (0.34)	14.4 (0.46)	12.8 (1.36)	<.0001
Any cardiovascular disease (% yes) ^a^	51.2 (0.17)	48.4 (0.80)	47.3 (1.28)	42.3 (0.43)	41.7 (0.66)	41.0 (2.23)	<.0001
Obesity (% yes) ^b^	31.6 (0.15)	26.5 (0.67)	31.2 (1.02)	33.8 (0.43)	10.8 (0.42)	27.7 (2.07)	<.0001
Serious psychological distress (% yes)	3.3 (0.06)	3.3 (0.28)	1.8 (0.29)	4.8 (0.19)	2.0 (0.19)	8.2 (1.31)	<.0001
Currently drink alcohol (% yes)	62.7 (0.22)	67.5 (0.72)	45.2 (1.16)	45.3 (0.46)	39.3 (0.73)	52.0 (2.42)	<.0001
Current smoker (% yes)	17.2 (0.13)	13.3 (0.54)	6.4 (0.49)	10.9 (0.27)	8.6 (0.38)	14.7 (1.63)	<.0001
Exercise ≥ 5 times/wk (% no)	68.8 (0.20)	67.6 (0.73)	76.7 (0.95)	77.4 (0.38)	67.7 (0.68)	72.0 (2.10)	<.0001
*Foreign-born only*							
Less than 15 y in United States	—	12.1 (0.63)	21.6 (1.05)	16.6 (0.37)	22.0 (0.60)	20.8 (1.80)	<.0001
U.S. citizen (% no)	—	21.2 (0.67)	29.5 (1.05)	41.8 (0.56)	24.8 (0.61)	20.0 (1.82)	<.0001

*Notes*: GED = General Education Development; NH = non-Hispanic; NHIS = National Health Interview Survey.

^a^ Cardiovascular disease determined by participants reporting ever had hypertension, coronary heart disease, angina, heart attack, stroke, or any other type of heart condition. ^b^ Obesity determined by a body mass index ≥30.

### Age- and Sex-Adjusted Prevalence

The age- and sex-adjusted prevalence of cognitive impairment is reported in Table 2. The prevalence of cognitive impairment was higher among Arab American immigrants (9.7%) compared to Hispanic (8.2%), non-Hispanic Asian (7.2%), non-Hispanic White (7.4%), and non-Hispanic Black (6.9%) immigrants, and U.S.-born non-Hispanic Whites (7.3%) (*p* < .0001).

**Table 2.** Age- and Sex-Adjusted Prevalence of Cognitive Impairment Among U.S.-Born and Immigrant Adults, NHIS 2000–2017, *N* = 228 985

	U.S.-Born			Foreign-Born			
	NH Whites, *n* = 189 129	NH Whites, *n* = 6048	NH Blacks, *n* = 3187	Hispanics, *n* = 21 367	NH Asians, *n* = 8467	Arab Americans, *n* = 787	
Variable	Weighted % (*SE*)	Weighted % (*SE*)	Weighted % (*SE*)	Weighted % (*SE*)	Weighted % (*SE*)	Weighted % (*SE*)	*p*-Value
Cognitive impairment	7.3 (0.00)	7.4 (0.00)	6.9 (0.00)	8.2 (0.00)	7.2 (0.00)	9.7 (0.01)	<.0001

*Note*: NH = non-Hispanic; NHIS = National Health Interview Survey.

### Logistic Regression Results

Crude and multivariable logistic regression results are reported in Table 3. In crude models, Arab American immigrants had 1.24 times greater odds (95% CI = 0.90, 1.73) of reporting cognitive impairment compared to U.S.-born Whites. Although results were not statistically significant in this crude model or Models 3 through 7, significant differences were found when adjusting for age and sex (Model 2; OR = 1.41; 95% CI = 1.02, 1.94). When we compared Arab American immigrants to non-Hispanic White immigrants, no statistically significant results were obtained.

**Table 3.** Crude and Adjusted Odds Ratios (95% confidence intervals) for Cognitive Impairment Among U.S.-Born and Immigrant Adults, NHIS 2000–2017, *N* = 228 985

Variable	Model 1^a^	Model 2^b^	Model 3^c^	Model 4^d^	Model 5^e^	Model 6^f^	Model 7^g^
Cognitive impairment							
U.S.-born NHW	1.00	1.00	1.00	1.00	1.00	1.00	—
Arab American immigrants	1.24 (0.90, 1.73)	1.41 (1.02, 1.94)	1.24 (0.87, 1.75)	1.12 (0.79, 1.59)	1.16 (0.82, 1.65)	1.13 (0.79, 1.60)	—
Foreign-born NHW	1.00	1.00	1.00	1.00	1.00	1.00	1.00
Arab American immigrants	1.14 (0.81, 1.60)	1.37 (0.99, 1.91)	1.20 (0.83, 1.72)	1.09 (0.76, 1.57)	1.10 (0.76, 1.59)	1.06 (0.73, 1.53)	1.06 (0.73, 1.53)

*Notes*: NHIS = National Health Interview Survey; NHW = non-Hispanic White.

^a^Unadjusted (Model 1); U.S.- and non-Hispanic White immigrants are reference groups. ^b^Adjusted for demographics (sex and age) (Model 2). ^c^Adjusted for Model 2 plus socioeconomic status (education, below poverty level, and employment) (Model 3). ^d^Adjusted for Model 3 plus comorbidity (diagnosed with heart disease, diabetes, obesity, and serious psychological distress) (Model 4). ^e^Adjusted for Model 4 plus behavioral risk factors (exercise, smoking, and drinking) (Model 5). ^f^Adjusted for Model 5 plus marital status and living alone (Model 6). ^g^Adjusted for Model 6 plus acculturation proxies (years in United States and citizenship status) for non-Hispanic White immigrants only (Model 7).

Our sensitivity analyses results are presented in Supplementary Table 1 (ages 55 and older) and Supplementary Table 2 (ages 65 and older). All confidence intervals overlapped with our initial findings, except for the crude results for adults ages 45 and older (OR = 1.24; 95% CI = 0.90, 1.73) and analysis limited to adults aged 65 and older (OR = 2.66, 95% CI = 1.77, 4.00).

## Discussion and Implications

The goals of this study were 2-fold: (a) to estimate and compare the age- and sex-adjusted prevalence of cognitive impairment among Arab American immigrants with other racial and ethnic groups; and (b) to examine the associations between cognitive impairment and race, ethnicity, and nativity status while adjusting for potential confounders. The indication of cognitive impairment was higher, at 9.7%, for Arab American immigrants compared to any other racial and ethnic group. This is the first study to undertake these analyses; therefore, we are unable to compare our finding to other studies of Arab Americans and cognitive impairment. Interestingly, if Arab Americans were not disaggregated from the non-Hispanic White population, it may have appeared that the prevalence of cognitive impairment was approximately 7.4% for Arab Americans.

There are several reasons that may explain the higher prevalence (9.7%) of cognitive impairment among Arab Americans. One is that many Arab Americans, especially in the last 10 years, have immigrated to the United States from war torn countries, perhaps as refugees (24). The toll of this process has greatly affected their mental health, as was observed with the study on serious psychological distress (19). A second factor that may have influenced the results is how the questions about cognitive impairment were translated and interpreted by Arab American respondents. Many respondents answered in English; however, the meaning of the words and questions may have varied between Arab Americans and other racial and ethnic groups. To our knowledge, valid and reliable questionnaires to assess cognitive impairment are not available in the Arabic language, and this is a worthwhile next step to pursue. It is also possible that the prevalence estimate of cognitive impairment among Arab Americans in this study was underestimated due to the limited number of languages in which the NHIS was available (i.e., English and Spanish). Arabic-speaking individuals, who may have had an even higher prevalence of cognitive impairment, were less likely to participate. One study showed that monolingual Spanish speakers have less education and lower cognitive performance than Spanish–English bilinguals in a sample of Hispanics (25). Arab Americans may display this pattern and further studies are needed to confirm this.

A noteworthy comparison is the findings from the current study and the study by Moon and colleagues (2). The current study demonstrated that Arab American immigrants had a higher prevalence of cognitive impairment compared to non-Hispanic White immigrants. The study by Moon and colleagues showed that non-Hispanic Black immigrants had a higher prevalence of dementia compared to U.S.-born non-Hispanic Blacks. While our study was not able to disaggregate U.S.-born Arab Americans from the non-Hispanic White group, we can hypothesize (given the lower prevalence of cognitive impairment among U.S.-born non-Hispanic Whites) that the prevalence of cognitive impairment was lower for U.S.-born Arab Americans compared to Arab American immigrants. Taken in context, it appears that this can be explained by cultural, social, and economic similarities between Arab American and non-Hispanic Black immigrants in the United States. Culturally, Arab American (26) and non-Hispanic Black families (27) tend to care for the older relatives at home. Socially, in various parts of the United States, Arab Americans and non-Hispanic Blacks tend to reside in proximate neighborhoods (28), especially those of lower socioeconomic status. This social context may contribute to possible similarities that might be observed between Arab American and non-Hispanic Black immigrants. Also, discrimination may play a role in the parallel patterns that could be observed between the 2 groups. For example, some Arab Americans have a darker complexion, wear religious garb, speak Arabic, etc., which would make them more noticeable compared to Arab Americans who can “pass for White.” This discrimination might lead to unequal access to health care and treatment (29).

The second main finding from this study is certain variables affect the association between race, ethnicity, nativity status, and cognitive impairment indicators. Only when adjusting for age and sex did we observe a statistically significant difference in indicators of cognitive impairment prevalence between Arab Americans and non-Hispanic Whites. This pattern of results likely reflects the fact that Arab Americans in this sample were younger than non-Hispanic Whites, perhaps because the oldest Arab Americans were more likely to be monolingual Arabic speakers. Only when “equalizing” the sample so that age was similar across groups did we notice a statistically significant disparity between Arab Americans and non-Hispanic Whites on cognitive impairment. With regard to sex, the sample for the study had a greater proportion of Arab American men (53.6%) compared to other groups, all of which were under 48%. This could signify the immigration pattern of Arab Americans, where men enter the United States first and their family follows. This pattern might even be more pronounced among the refugees from the Middle East. With higher social support and networks, Arab American women may be less likely to suffer from cognitive impairment compared to Arab American men.

The difference between Arab American and non-Hispanic White immigrants became nonsignificant when socioeconomic variables were added to the model, suggesting that while Arab American immigrants may share characteristics with other immigrants to the United States, they also face unique challenges that may have implications for late-life cognitive health. While we are just beginning to discover these trends, the literature is consistent in showing, when compared to non-Hispanic Whites, Arab Americans tend to be younger, male, have higher education, and are more likely to be employed, but more likely to live in poverty. Indeed, compared to both U.S.-born and non-Hispanic White immigrants, Arab American immigrants in the current sample were more likely to have a college degree and more likely to be employed, but they were also more likely to have less than a high school degree and more likely to live in poverty. To better understand how these variables affect cognitive impairment disparities involving Arab Americans, it is important to obtain more granular ways of collecting data on socioeconomic status, for example. In addition, using qualitative methodology will be important to ensure that the meaning of words and questions are accurately represented in this population.

The significant difference in cognitive impairment prevalence between Arab American immigrants and U.S.-born non-Hispanic Whites became nonsignificant when physical and mental health (i.e., heart disease, diabetes, obesity, serious psychological distress) was added to the model. This pattern of results suggests that these health disparities, which have been documented for Arab Americans (6,8,21) may, in part, drive cognitive disparities. Compared to both U.S.-born and non-Hispanic White immigrants, Arab American immigrants in this study showed higher rates of diabetes and psychological distress, both of which have been associated with higher risk of developing dementia (30,31). In addition, Arab American immigrants in this study were more likely to report smoking than non-Hispanic White immigrants, and smoking is a risk factor for cognitive decline (32). Arab Americans in the current study were more likely to report diabetes and serious psychological distress, compared to both U.S.-born and non-Hispanic White immigrants. They were also more likely to report current smoking compared to all other immigrant groups, but not U.S.-born Whites. Focusing on health behaviors, chronic diseases, and mental disorders among Arab Americans may help to reduce cognitive impairment disparities.

### Strengths and Limitations

The strengths of this study are the use of nationally representative data, a large sample size, the ability to disaggregate the Arab American population from non-Hispanic Whites, and the inclusion of questions to assess cognitive impairment. The NHIS is the only national health study where Arab Americans can be identified, because the study includes a question on place of birth. An additional strength of this study is the use of sensitivity analysis to confirm our findings with different age cutoffs. Although our crude results comparing Arab American immigrants and U.S.-born non-Hispanic Whites differed among our samples aged 45 and older (OR = 1.24; 95% CI = 0.90, 1.73) and 65 and older (OR = 2.66; 95% CI = 1.77, 4.00), significant results for adults aged 65 and older should be interpreted with caution due to the smaller sample size of Arab American immigrants with self-reported cognitive impairment in this age group (65+ years, *n* = 58; 55+ years, *n* = 71; 45+ years, *n* = 87).

The drawback is the NHIS includes identification of only Arab American immigrants, and not Arab Americans born in the United States. If they were born in the United States, they are still included in the non-Hispanic White category, and we are unable to disaggregate them. Thus, we may have underestimated the disparity between Arab American immigrants and U.S.-born Whites because U.S.-born Arab Americans were included in the comparison group. While the NHIS includes 2 questions related to cognitive impairment, these are not comprehensive, and objective assessments would be needed to obtain gold-standard diagnoses of cognitive impairment. We used the question that captures a more established measure of cognitive impairment, that has been used by other researchers (1), and that yielded a more reliable sample size. The questions are self-reported, and a diagnosis from a health care provider would be more valid and reliable. Lastly, while the NHIS is a nationally representative survey, this does not mean that the Arab American subsample is representative of the larger Arab American population. The fact that the NHIS is the closest we can get to obtaining nationally representative data for Arab Americans underscores the importance of our work, which increases the visibility of this population for health researchers and highlights the need for more representative sampling of this understudied group. An additional limitation is who is reporting on cognitive impairment. While most responses were collected directly from participants, a proxy was used if one was needed. This approach introduces additional variability to how the question is translated and answered.

In conclusion, this is the first study to provide estimates of indicators of cognitive impairment among Arab American immigrants. One interesting observation that is emerging from the literature on Arab American health is that Arab Americans do not align with the “healthy migrant” hypothesis (5). This hypothesis portends that immigrants tend to be healthier than their U.S.-born counterparts. The immigration process and journey can be physically, mentally, and financially challenging; therefore, only individuals who are resilient and “hearty” may be able to immigrate to another country. The health literature on Arab Americans demonstrates that they do not fit this pattern. In fact, Arab immigrants to the United States tend to have poorer health compared to U.S.-born Arab Americans. Even though these individuals may not be “healthy” in their country of origin, the political predicaments may have forced them to leave as refugees or asylees, as opposed to their own free will. From a policy perspective, both state and national level efforts need to include an ethnic identifier for Arab Americans so that health and health behavior patterns can more easily be observed and used in prevention and intervention efforts.

## Supplementary Material

igaa058_suppl_Supplementary_Tables_S1-S2
