# Supplementary material for: Prevalence of Self-reported Cognitive Impairment Among Arab American Immigrants in the United States
Source: Innov Aging. 2020 Nov 24;5(1):igaa058. doi: 10.1093/geroni/igaa058 (PMC7788314; doi:10.1093/geroni/igaa058)
Supplement: igaa058_suppl_Supplementary_Tables_S1-S2 [file igaa058_suppl_supplementary_tables_s1-s2.docx]

Supplemental Table 1. *Crude and adjusted odds ratios (95% confidence intervals) for cognitive impairment among US-born and immigrant adults ages 55 and older, NHIS 2000-2017, N=154,456*

| **Variable** | **Model 1 ^a^** | **Model 2** **^b^** | **Model 3 ^c^** | **Model 4 ^d^** | **Model 5 ^e^** | **Model 6 ^f^** | **Model 7 ^g^** |
| --- | --- | --- | --- | --- | --- | --- | --- |
| **Cognitive Impairment** | | |  |  |  |  |  |
| US-born NHW | 1.00 | 1.00 | 1.00 | 1.00 | 1.00 | 1.00 | -- |
| Arab American Immigrants | 1.64 (1.15, 2.33) | 1.89 (1.33, 2.68) | 1.70 (1.16, 2.49) | 1.57 (1.07, 2.32) | 1.70 (1.14, 2.53) | 1.59 (1.06, 2.39) | -- |
| Foreign-born NHW  Arab American Immigrants | 1.00  1.42 (0.98, 2.05) | 1.00  1.72 (1.20, 2.48) | 1.00  1.53 (1.02, 2.30) | 1.00  1.47 (0.97, 2.22) | 1.00  1.52 (1.00, 2.32) | 1.00  1.42 (0.92, 2.20) | 1.00  1.42 (0.92, 2.20) |

*Notes.* US = United States; NHIS = National Health Interview Survey; NHW = Non-Hispanic white.

^a^ Unadjusted (model 1); US- and non-Hispanic white immigrants are reference groups.

^b^ Adjusted for demographics (sex, age) (model 2)

^c^ Adjusted for model 2 plus socioeconomic status (education, below poverty level, employment) (model 3)

^d^ Adjusted for model 3 plus comorbidity (diagnosed with heart disease, diabetes, obesity, SPD) (model 4)

^e^ Adjusted for model 4 plus behavioral risk factors (exercise, smoking, drinking) (model 5)

^f^ Adjusted for model 5 plus marital status and living alone (model 6)

^g^ Adjusted for model 6 plus acculturation proxies (years in US and citizenship status) for non-Hispanic white immigrants only (model 7)

Supplemental Table 2. *Crude and adjusted odds ratios (95% confidence intervals) for cognitive impairment among US-born and immigrant adults ages 65 and older, NHIS 2000-2017, N=95,376*

|  | **Model 1**^a^ | **Model 2** ^b^ | **Model 3** ^c^ | **Model 4** ^d^ | **Model 5** ^e^ | **Model 6** ^f^ | **Model 7** ^g^ |
| --- | --- | --- | --- | --- | --- | --- | --- |
| **Cognitive Impairment** | | |  |  |  |  |  |
| US-born NHW | 1.00 | 1.00 | 1.00 | 1.00 | 1.00 | 1.00 | -- |
| FB Arab Americans | 2.66 (1.77, 4.00) | 3.81 (1.87, 4.23) | 2.54 (1.60, 4.05) | 2.36 (1.49, 3.75) | 2.52 (1.57, 4.08) | 2.39 (1.47, 3.88) | -- |
|  |  |  |  |  |  |  |  |
| Foreign-born NHW  FB Arab Americans | 1.00  2.15 (1.42, 3.25) | 1.00  2.37 (1.57, 3.58) | 1.00  2.18 (1.36, 3.49) | 1.00  2.14 (1.34, 3.44) | 1.00  2.27 (1.41, 3.68) | 1.00  2.16 (1.32, 3.51) | 1.00  2.13 (1.31, 3.48) |

*Notes.* US = United States; NHIS = National Health Interview Survey; NHW = Non-Hispanic white.

^a^ Unadjusted (model 1); US- and non-Hispanic white immigrants are reference groups.

^b^ Adjusted for demographics (sex, age) (model 2)

^c^ Adjusted for model 2 plus socioeconomic status (education, below poverty level, employment) (model 3)

^d^ Adjusted for model 3 plus comorbidity (diagnosed with heart disease, diabetes, obesity, SPD) (model 4)

^e^ Adjusted for model 4 plus behavioral risk factors (exercise, smoking, drinking) (model 5)

^f^ Adjusted for model 5 plus marital status and living alone (model 6)

^g^ Adjusted for model 6 plus acculturation characteristics (years in US and citizenship status) for non-Hispanic white immigrants only (model 7)
